# Transcriptional profile of *Mycobacterium tuberculosis* infection in people living with HIV

**DOI:** 10.1016/j.isci.2024.111228

**Published:** 2024-10-21

**Authors:** Burcu Tepekule, Lisa Jörimann, Corinne D. Schenkel, Lennart Opitz, Jasmin Tschumi, Rebekka Wolfensberger, Kathrin Neumann, Katharina Kusejko, Marius Zeeb, Lucas Boeck, Marisa Kälin, Julia Notter, Hansjakob Furrer, Matthias Hoffmann, Hans H. Hirsch, Alexandra Calmy, Matthias Cavassini, Niklaus D. Labhardt, Enos Bernasconi, Gabriela Oesch, Karin J. Metzner, Dominique L. Braun, Huldrych F. Günthard, Roger D. Kouyos, Fergal Duffy, Johannes Nemeth, the Swiss HIV Cohort Study

**Affiliations:** 1 Department of Infectious Diseases and Hospital Epidemiology, University Hospital Zurich, Zurich, Switzerland; 2 Institute of Medical Virology, University of Zurich, Zurich, Switzerland; 3 Department of Ecology and Evolutionary Biology, Princeton University, Princeton, NJ, USA; 4 Functional Genomics Center Zurich, Swiss Federal Institute of Technology and University of Zurich, Zurich, Switzerland; 5 Department of Biomedicine, University of Basel, Basel, Switzerland; 6 Division of Infectious Diseases and Hospital Epidemiology, Cantonal Hospital St Gallen, St. Gallen, Switzerland; 7 Department of Infectious Diseases, Inselspital, Bern University Hospital, University of Bern, Bern, Switzerland; 8 Division of Infectious Diseases and Hospital Epidemiology, Cantonal Hospital Olten, Olten, Switzerland; 9 Division of Infectious Diseases and Hospital Epidemiology, University Hospital Basel, Basel, Switzerland; 10 Clinical Virology, Laboratory Medicine, University Hospital Basel, Basel, Switzerland; 11 Department Biomedicine, Transplantation and Clinical Virology, University of Basel, Basel, Switzerland; 12 Division of Infectious Diseases, University Hospital Geneva, University of Geneva, Geneva, Switzerland; 13 Division of Infectious Diseases, University Hospital Lausanne, University of Lausanne, Lausanne, Switzerland; 14 Division Clinical Epidemiology, Department of Clinical Research, University Hospital Basel, Basel, Switzerland; 15 University of Basel, Basel, Switzerland; 16 Division of Infectious Diseases, Ente Ospedaliero Cantonale, Lugano, Switzerland; 17 University of Geneva and University of Southern Switzerland, Lugano, Switzerland; 18 Department of Child Neurology, Inselspital, Bern University Hospital, University of Bern, Bern, Switzerland; 19 Seattle Children’s Research Institute, Seattle, WA, USA

**Keywords:** Health sciences, Microbiology, Transcriptomics

## Abstract

In people with HIV-1 (PWH), *Mycobacterium tuberculosis* (MTB) infection poses a significant threat. While active tuberculosis (TB) accelerates immunodeficiency, the interaction between MTB and HIV-1 during asymptomatic phases remains unclear. Analysis of peripheral blood mononuclear cells (PBMC) transcriptomic profiles in PWH, with and without controlled viral loads, revealed distinct clustering in MTB-infected individuals. Functional annotation identified alterations in IL-6, TNF, and KRAS pathways. Notably, MTB-related genes displayed an inverse correlation with HIV-1 viremia, at both individual and signature score levels. These findings suggest that MTB infection in PWH induces a shift in immune system activation, inversely related to HIV-1 viral load. These results may explain the observed enhanced antiretroviral control in MTB-infected PWH. This study highlights the complex interplay between MTB and HIV-1, emphasizing the importance of understanding their interaction for managing co-infections in this population.

## Highlights

• MTB infection perturbs the PBMC transcriptome in PWH, independent of MTB re-exposure
• MTB infection shifts baseline inflammation, affecting IL-6, TNF-alpha, and KRAS
• MTB-associated perturbations correlate with improved retroviral control
• MTB-associated perturbations are lost during progression to active tuberculosis

## Graphical abstract

**Figure:**
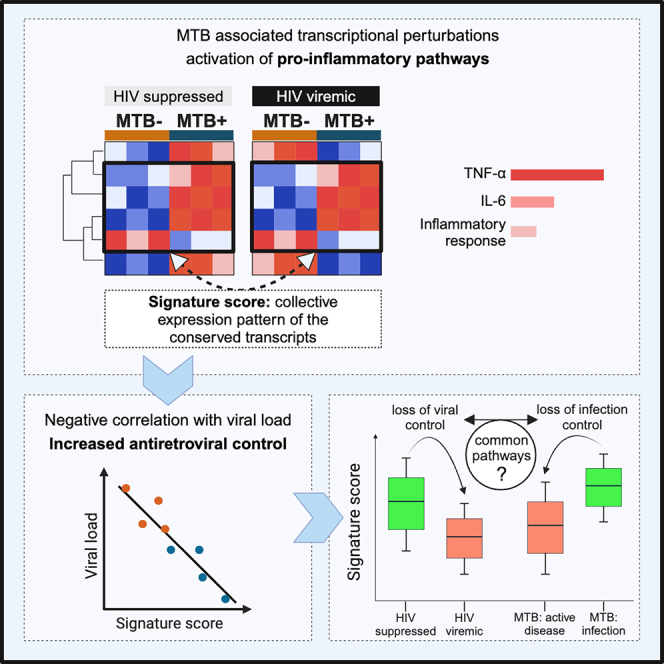

## Introduction

Globally, HIV-1 remains one of the most important risk factors for the development of active tuberculosis (TB).^1^^,^^2^^,^^3^ Individuals with HIV-1 and active TB are at risk for earlier TB dissemination, increased risk of faster progression, and TB drug resistance.^1^

However, active TB represents only the most severe manifestation within the spectrum of *Mycobacterium tuberculosis* (MTB) infection. There are ample efforts to advance the field beyond the binary concept of infection versus disease into a more granular description of the disease spectrum.^4^^,^^5^ Irrespective of HIV status, only a minority of individuals exposed to MTB will develop active TB: while the exact numbers remain uncertain, it is well accepted that less than 10% of MTB-infected HIV-uninfected individuals ever develop active disease.^1^^,^^2^ Similarly, only a minority of people with HIV-1 (PWH) exposed to MTB will progress to active TB.^2^^,^^6^

In this work, we hypothesized that exposure to MTB does not only induce an antigen specific T cell response but also impacts the activation state of the innate immune system analogous to the trained immunity phenotype described after BCG vaccination.^7^^,^^8^ In PWH-1, MTB infection but not active TB was associated with significantly reduced HIV-1 viral set point and with a lower frequency of common opportunistic infections.^9^ Also, MTB infection is associated with increased risk of developing diabetes mellitus and increased cardiovascular risk, suggesting there is an evolutionary trade-off for the immune modulatory effects of MTB infection.^10^^,^^11^ In this study, we investigated whether changes in the immune system induced by MTB infection, as predicted from epidemiological observations, can be detected in peripheral blood in PWH. We also explored their potential relationship with the HIV-1 viral set point, considering the significant and consistent impacts observed at the population level.^9^

Until now, the sole method of verifying exposure to MTB in clinical practice in the absence of symptomatic TB has been through the identification of MTB specific T cells either by stimulating T cells intradermally (tuberculin skin test) or circulation, known as interferon gamma release assays (IGRA). However, this approach has significant limitations. The presence of MTB specific T cells could indicate one of three possibilities. Firstly, it could suggest the presence of bacteria that are in a state of reversible latency and have the potential to cause disease. Secondly, it could indicate bacteria that are in a state of practically “irreversible” latency, requiring an excessive and unsustainable amount of immunosuppression to become active again, incompatible with the host’s survival. Lastly, it could signify a memory T cell response following the eradication of the infection.^12^ Additionally, the efficacy of IGRA relies on the functionality of T cells, which can be impaired due to the replication of HIV-1.^6^

## Results

### MTB infection perturbs the transcriptome of PWH with suppressed viral load

To investigate the impact of MTB infection on the transcriptome of PWH, we conducted a detailed analysis using peripheral blood mononuclear cells (PBMC). 78% of samples coinciding with the TB testing date. Total follow up was median 78.5 months after MTB testing (range 9.7–244.5 months). We focused PWH with a suppressed viral load to adjust for the potential effects of a replicating HIV-1 infection on the transcriptome. The algorithm matched 22 PWH in pairs based on their CD4 cell count, ethnicity, and sex to minimize further confounding variables (Table 1).

**Table 1:** Characteristics of suppressed PWH

	MTB+ HIV^supp^	MTB- HIV^supp^	all HIV^supp^	*p* value
	9	9	18	
CD4 (median [IQR])	480 [464, 549]	482 [415, 547]	481 [454, 548]	0.938
Viral load (median [IQR])	0 [0, 0]	0 [0, 0]	0 [0, 0]	–
TB test type (%)				
TST	2 (22.2)	3 (33.3)	5 (27.7)	0.624
IGRA	7 (77.7)	6 (66.6)	13 (72.2)	–
Sex (%)				
Male	8 (88.8)	8 (88.8)	16 (88.8)	1
Female	1 (11.1)	2 (11.1)	2 (11.1)	–
Ethnicity (%)				
White	7 (77.7)	7 (77.7)	14 (77.7)	1
Black	1 (11.1)	1 (11.1)	2 (11.1)	–
Hispano-american	1 (11.1)	1 (11.1)	2 (11.1)	–

Shown are median and interquartile range or % for MTB+ HIV^supp^ (*n* = 9), MTB- HIV^supp^ (*n* = 9) and all HIV^supp^ (*n* = 18) with corresponding *p* values from two sample t-tests. TST, Tuberculin skin test; IGRA, Interferon gamma release assay. All patients are on antiretroviral therapy (ART).

**Figure 1:**
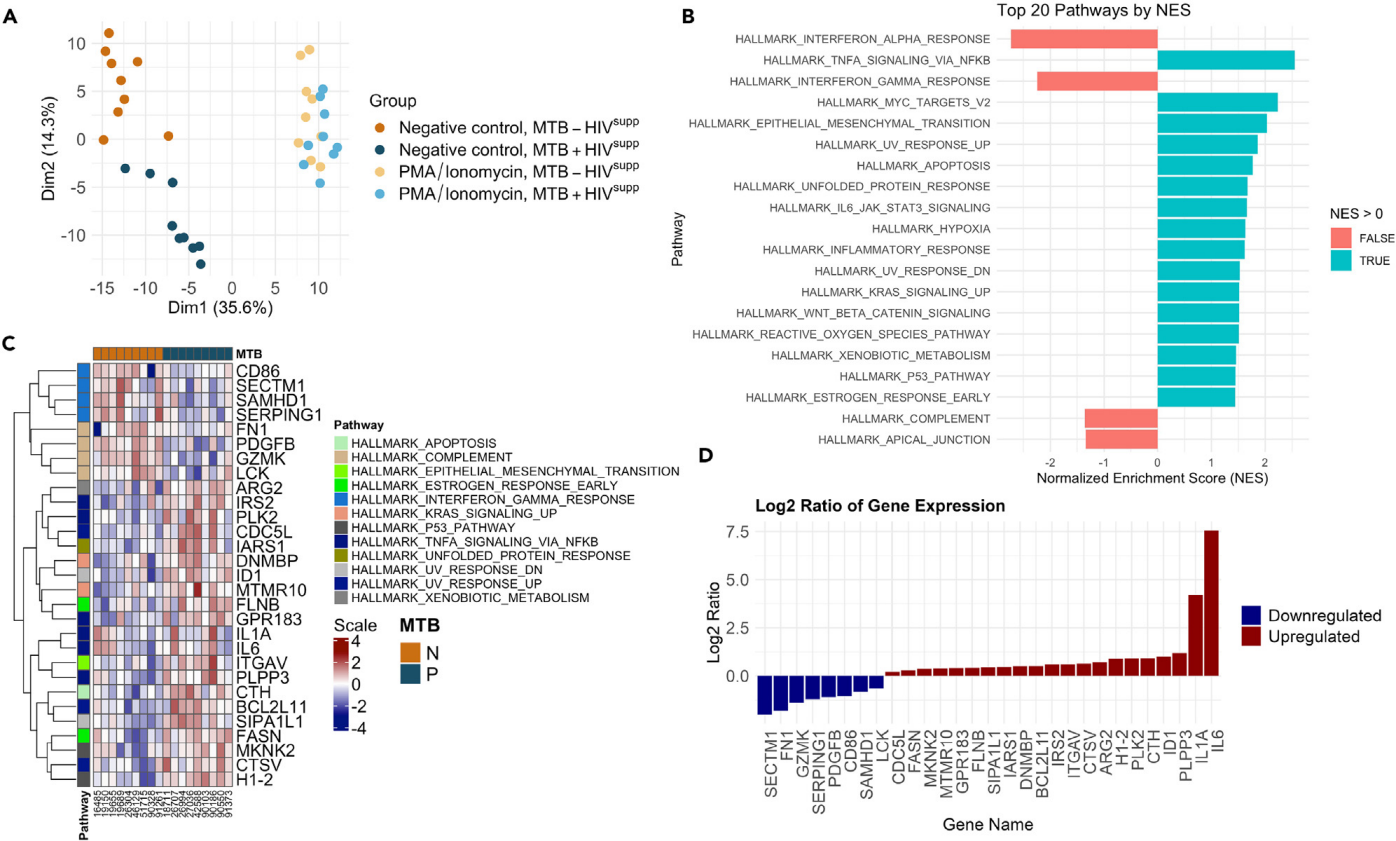
MTB Perturbations in suppressed PWH (A) Dimensionality reduction and clustering analysis for HIV-suppressed individuals with and without MTB infection (MTB+ HIV^supp^ and MTB- HIV^supp^). Genes with a *p* value lower than 0.01 are used for the analysis, corresponding to 293 genes out of the 21493 analyzed. In MTB+ patients compared to MTB- patients, 126 out of 293 genes were upregulated and 167 were downregulated. (B) First 20 pathways with the highest normalized enrichment score (NES) based on the gene-set enrichment analysis (GSEA). (C) Heatmap displaying the leading edge genes of these pathways filtered by *p* value <0.01. The color scale ranges from dark blue (representing downregulated genes) to dark red (representing upregulated genes), with lighter tones indicating intermediate values. Rows represent genes, and columns represent samples, annotated by MTB status (N for negative, P for positive). The data are log2-transformed and clustered by genes. The scale bar indicates log2 expression levels ranging from −4 to 4. (D) Fold change (Log2 ratios) of the differentially expressed genes.

Our analysis revealed distinct transcriptome profiles between HIV-suppressed individuals with and without MTB infection (MTB+ HIV^supp^ and MTB- HIV^supp^), particularly in non-activated PBMCs, as shown by dimensionality reduction and clustering (Figure 1A). The uniform response to PMA/Ionomycin stimulation across all samples indicated that all the samples contained viable cells.

Using gene set enrichment analysis (GSEA), we ranked the pathways with the highest normalized enrichment score (NES) (Figure 1B), displayed the leading edge genes of these pathways in a heatmap (Figure 1C) and showed the fold change of the differentially expressed genes (Figure 1D). Flow cytometry analysis revealed no significant differences in the main PBMC subpopulations, suggesting that the transcriptomic changes are not due to variations in cell type frequencies (Figure S1A, related to Figures 1 and S1B).

### MTB infection perturbs the transcriptome of PWH with replicating HIV-1 virus

**Figure 2:**
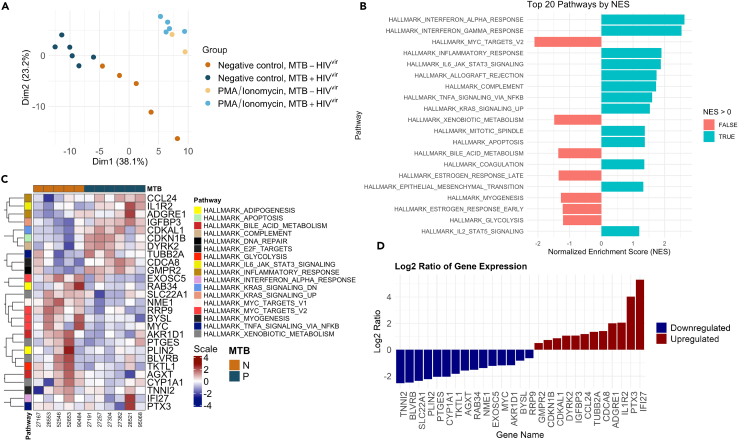
MTB Perturbations in viremic PWH (A) Dimensionality reduction and clustering analysis for HIV-viremic individuals with and without MTB infection (MTB+ HIV^vir^ and MTB- HIV^vir^). Genes with a *p* value lower than 0.01 are used for the analysis, corresponding to 166 genes out of the 21467 analyzed. In MTB+ patients compared to MTB- patients, 68 out of 166 genes were upregulated and 98 were downregulated. (B) First 20 pathways with the highest normalized enrichment score (NES) based on the gene-set enrichment analysis (GSEA). (C) Heatmap displaying the leading edge genes of these pathways filtered by *p* value <0.01. The color scale ranges from dark blue (representing downregulated genes) to dark red (representing upregulated genes), with lighter tones indicating intermediate values. Rows represent genes, and columns represent samples, annotated by MTB status (N for negative, P for positive). The data are log2-transformed and clustered by genes. The scale bar indicates log2 expression levels ranging from −4 to 4. (D) Fold change (Log2 ratios) of the differentially expressed genes.

Next, we examined the impact of MTB infection on HIV viral load in viremic PWH. (Table 2). Consistent with our findings in suppressed PWH, dimensionality reduction and clustering analysis identified distinct transcriptomic profiles between MTB+ HIV^vir^ and MTB- HIV^vir^ (Figure 2A). The clear separation between the MTB+ and MTB- groups emphasizes the significant impact of MTB infection on the transcriptome, an effect that remained notably detectable despite the extensive perturbations caused by HIV-1 infection.^13^

**Table 2:** Characteristics of viremic PWH

	MTB+ HIV^vir^	MTB- HIV^vir^	all HIV^vir^	*p* value
	6	5	11	
CD4 (median [IQR])	534 [420, 676]	409 [337, 699]	453 [373, 698]	0.79
Viral load (median [IQR])	30802 [13710, 123422]	28427 [15137, 120000]	28427 [12048, 136674]	0.88
TB test type (%)				
IGRA	6 (100.0)	5 (100.0)	11 (100.0)	–
Sex (%)				
Male	5 (83.3)	5 (100.0)	10 (90.9)	0.36
Female	1 (16.6)	0 (0.0)	1 (9.1)	–
Ethnicity (%)				
White	6 (100.0)	5 (100.0)	11 (100.0)	–

Shown are median and interquartile range or % for MTB+ HIV^vir^ (*n* = 6), MTB- HIV^vir^ (*n* = 5) and all HIV^vir^ (*n* = 11) with corresponding *p* values from two sample t-tests. IGRA, Interferon gamma release assay. All patients are on antiretroviral therapy (ART).

We then investigated the transcriptional changes due to MTB infection in viremic PWH with GSEA and ranked the pathways with the highest NES (Figure 2B), displayed the leading edge genes of these pathways in a heatmap (Figure 2C) and showed the fold change of the differentially expressed genes (Figure 2D).

In sum, in subjects with MTB infection, a noteworthy and sustained upregulation was discerned in three hallmark pathways when compared to their non-infected counterparts. These pathways include tumor necrosis factor (TNF) signaling, KRAS signaling, and interleukin-6 (IL-6) signaling, irrespective of the occurrence of human immunodeficiency virus type 1 (HIV-1) viremia, as illustrated in Figures 1B and 2B. Flow cytometry analysis revealed no significant differences in the main PBMC subpopulations, suggesting that the transcriptomic changes are not due to variations in cell type frequencies (Figure S1B, related to Figure S1A; Figure 2).

### Testing the MTB-HIV-1 interaction on single gene level

At the level of molecular pathways, our findings indicate that MTB infection in PWH is linked to increased activation of pro-inflammatory pathways, regardless of the presence of HIV-1 viremia. Given the observation that MTB infection is correlated with a reduction in HIV-1 viral load,^9^ it is tempting to hypothesize that the heightened activity of these upregulated pathways may play a role in explaining this observed phenomenon.

**Figure 3:**
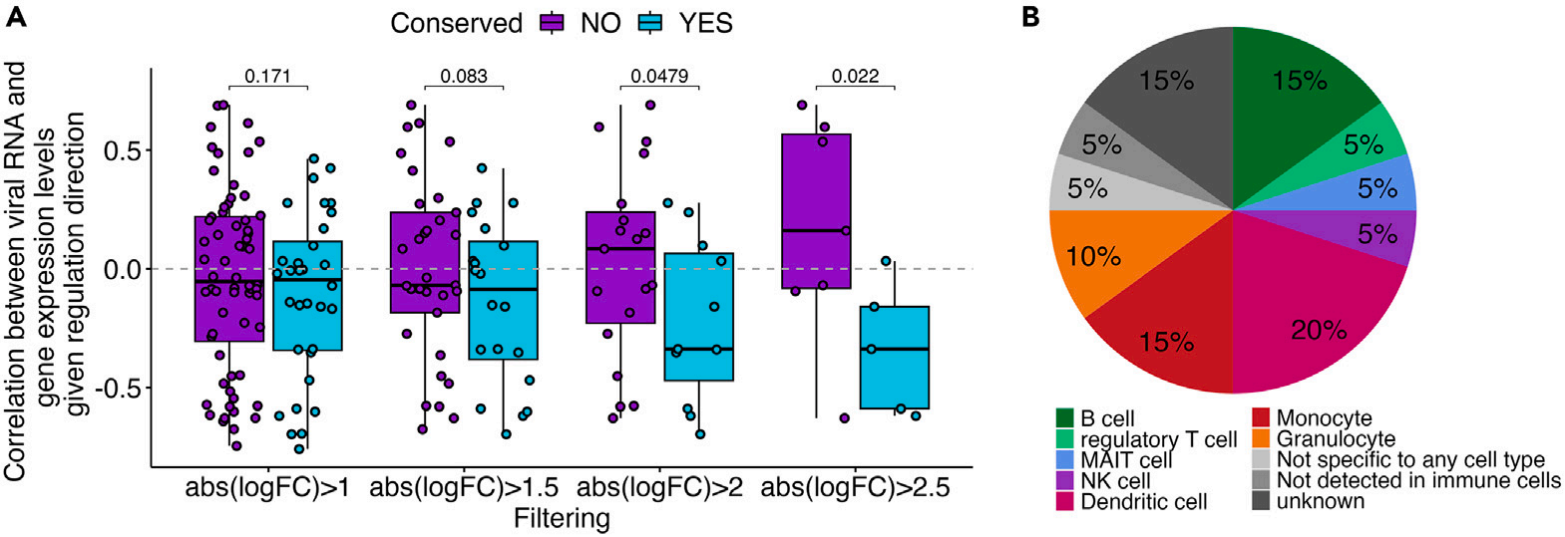
Correlation between HIV-1 viral load and the expression levels of conserved transcripts (A) Correlation between HIV-1 viral load and the expression levels of conserved transcripts across different fold-change thresholds. Data are represented as boxplots where the central line represents the median, and the boxes extend from the 25th to the 75th percentiles (interquartile range). Individual data points are plotted on top using jitter for clarity. A one-sided t-test was performed to compare groups, and *p* values are shown. (B) Percentages of different immune cells known to express the individual genes from the conserved transcript set with a negative correlation to viral load (20 transcripts out of 32), according to the human protein atlas.^14^

To test this association on a single gene level, we identified “conserved transcripts” as genes that were consistently differentially expressed in the same direction in MTB+ individuals compared to MTB- irrespective of HIV viremia status (full list in Table S1, related to Figures 1, 2, and 3). This categorization of genes identifies genes associated with MTB infection as a common denominator across HIV-1 viremic and HIV-1 suppressed patients.

Then, we examined the correlation between HIV-1 viral load and the expression levels of these genes across different fold-change thresholds (Figure 3A). We adjusted these correlations to account for the direction of gene regulation. For instance, if a gene is downregulated in MTB+ compared to MTB-, but its expression is inversely correlated with viral load, the correlation value would be inverted (multiplied by −1). Notably, as we raised the fold-change threshold—thereby focusing on genes with greater differences in expression according to the MTB status—we found a stronger and more statistically significant negative correlation with viral load, speaking to the strength of the effect.

To determine the probability of these correlations arising by chance, we employed a permutation test. This involved generating multiple random subsets from the pool of all transcripts and calculating the average correlation between the expression levels and the viral load in these randomized sets. We then compared this distribution to the correlations observed in our conserved transcripts. The results of the permutation test gave a *p* value of less than 2.2e-16 (Figure S3, related to Figure 3), demonstrating that the negative correlation between the expression of conserved transcripts and viral load was highly unlikely to occur by chance.

To understand which cells are most actively engaged in this mitigating effect, we analyzed the proportions of different immune cells expressing these transcripts (Figure 3B). Figure 3B illustrates that 50% of the genes were linked to cells originating from the innate immune system, while 25% were associated with the adaptive immune system.

### Testing the MTB-HIV-1 interaction integrating the single gene information into a signature score

**Figure 4:**
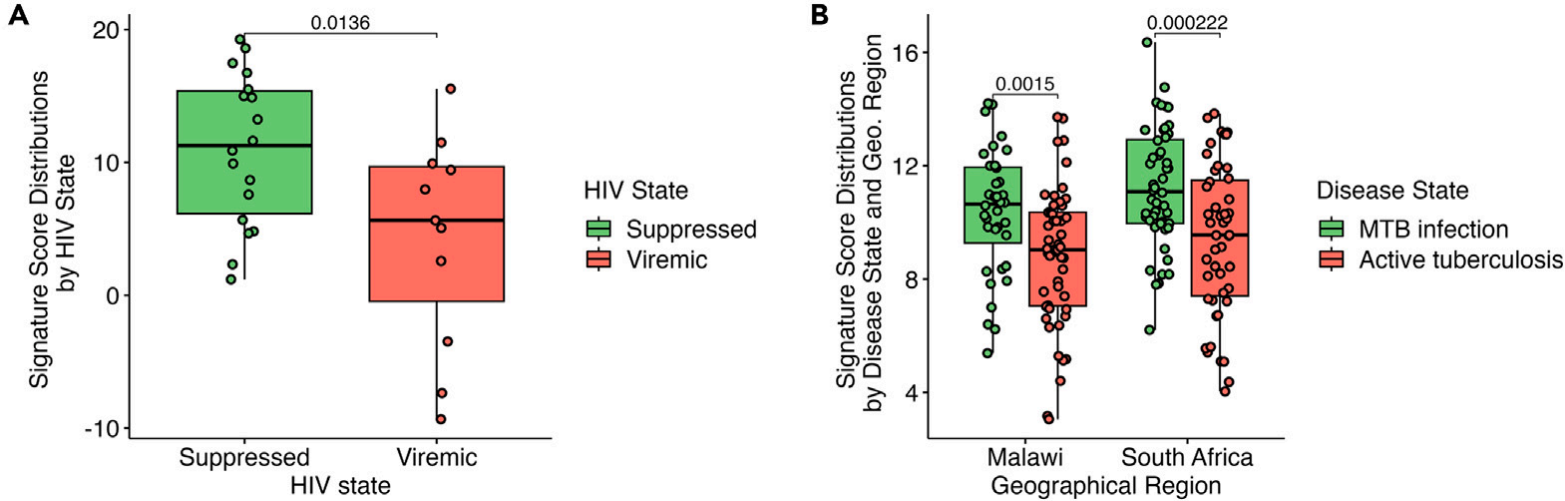
Signature score distributions for conserved transcripts (A) Signature score distributions of all conserved transcripts by HIV state. Data are represented as boxplots where the central line represents the median, and the boxes extend from the 25th to the 75th percentiles (interquartile range). Individual data points are plotted on top using jitter for clarity. A one-sided t-test was performed to compare groups, and *p* values are shown. (B) Signature Score Distributions in active TB. Signature score distributions including all 9 conserved transcripts that were present in our study and in the African cohort. Data are represented as boxplots where the central line represents the median, and the boxes extend from the 25th to the 75th percentiles (interquartile range). Individual data points are plotted on top using jitter for clarity. A one-sided t-test was performed to compare groups, and *p* values are shown.

To quantify the collective expression pattern of the conserved transcripts defined previously, we calculated the signature score^15^ using the 32 genes provided in Table S1, related to Figures 1, 2, and 3. Signature score is calculated by subtracting the average log10 expression of downregulated genes from the average log10 expression of upregulated genes across the set of conserved transcripts. We first compared this score between viremic and suppressed patients, suggesting a significant difference between the score values (Figure 4A). We then calculated the correlation of this signature score with the HIV-1 viral load in PWH, suggesting a negative correlation coefficient of −0.31. Given the small sample size, our analysis may be underpowered to detect small but biologically meaningful effects. While our findings indicate a weak negative correlation between the signature score and HIV viral load, the observed trend is consistent with the hypothesized role of conserved transcripts in influencing HIV control.

Previous studies, including our own, have shown that active TB is associated with increased HIV-1 viral load.^1^^,^^9^^,^^16^ We therefore hypothesized that increased antiretroviral control observed in asymptomatic MTB infection might be lost during active TB. To test this hypothesis, we focused on the “conserved transcripts” that are upregulated during asymptomatic MTB infection and inversely associated with HIV-1 viral set point, predicting these would be downregulated in active TB. We analyzed their expression in an independent cohort of PWH with asymptomatic MTB or active TB infection from Malawi and South Africa.^17^ Our analysis showed that PWH developing active TB had a significantly lower signature score compared to PWH MTB+ when calculated over these gene sets (Figure 4B). The lower signature scores observed in both viremic and active TB patients compared to those who are suppressed or asymptomatically infected with MTB support our hypothesis that losing control over MTB infection also affects key genes associated with retroviral control, as represented by the set of conserved transcripts in our results.

## Discussion

In this study, we have shown that MTB-specific T cell responses correlate with systemic transcriptional changes in PWH, independently of HIV-1 viral load. These MTB-associated transcriptional changes were inversely correlated with HIV-1 viremia, suggesting a potential causal link between MTB infection and improved control of HIV-1 replication.

The consistent upregulation of TNF alpha signaling via NF-κB and the IL6-JAK-STAT3 pathway in MTB infection irrespective of HIV-1 viremia indicates a sustained inflammatory response. An independent study found increased IL-6 and TNF-a levels on a protein level to be associated with MTB infection, further inducing confidence in our findings.^16^ The pro-inflammatory cytokines IL-6 and TNF-a have traditionally been associated with active disease in various cross-sectional studies comparing active TB patients to treated patients or healthy controls.^16^^,^^18^^,^^19^ Also, IL-6 levels have been shown to be predictors of unfavorable outcomes in patients with active TB.^19^ Conversely, IL-6 signaling has been hypothesized to be advantageous by inhibiting detrimental Type 1 Interferon responses.^20^ Our investigation adds a different aspect to IL-6 signaling during MTB infection: we demonstrate the activation of these very pathways in individuals who will remain free from active disease for years to decades—and this despite the absence of preventive treatment. Consequently, the detected pro-inflammatory response in our patients may indeed contribute to a protective immune response. If this protective immune response is sterilizing (clearing MTB infection) or contains viable bacteria for decades remains unclear.

Subsequently, our objective was to establish a correlation between alterations in the inflammatory state and potential heterologous effects, specifically focusing on HIV-1 control.^9^ We systematically examined these perturbations at the pathway, single gene, and signature score levels, unveiling an inverse relationship between the expressions of MTB infection associated genes and the efficacy of HIV-1 control. This notion of the baseline infection state influencing HIV-1 control is not unprecedented; for instance, TNF-a expression was documented to display a negative correlation with the HIV-1 latent reservoir in a recent cohort of individuals with HIV.^21^ It is noteworthy that our investigation contributes to the existing body of literature by emphasizing that these genes were defined by MTB infection status (and not, like in other investigations, by HIV-1 phenotype), therefore suggesting a heterologous mechanism.

It is important to recognize that the genes we identified in our study are not the sole determinants of HIV control, but rather exemplify the concept that certain features of the innate immune response induced by an asymptomatic MTB infection are consistent across various HIV viremic states. These genes are correlates of a broader immune modulation, suggesting that the MTB-infection associated alterations in the immune system—mediated through multiple potential mechanisms, including the proinflammatory pathways identified in our study—may contribute to a form of innate immune memory^22^^,^^23^ that confers a more generalized state of defense against HIV. While our focus on these conserved transcripts has provided valuable insights, they represent just one aspect of the complex interplay between MTB infection and HIV control. Future studies are needed to unravel the full spectrum of immune responses and identify additional genetic markers that contribute to the control of HIV replication.

Our findings indicate that the transcriptional changes associated with improved antiretroviral immunity in MTB-infected individuals are lost during active TB. In PWH from Malawi and South Africa, we assessed the expression of genes that are upregulated during asymptomatic MTB infection and inversely associated with HIV-1 viral set point in our initial analysis, and found a notable decrease in their expression among individuals with active TB compared to those with asymptomatic MTB infection, supporting the hypothesis that retroviral control is lost during active TB.

Our study has two major strengths. Firstly, the Swiss HIV Cohort Study (SHCS) provides a unique advantage in allowing us to meticulously match individuals with and without MTB infection, offering a high level of granularity for the clinical phenotypes and the ability to match individual patients for our analysis. Secondly, the study stands out for its exceptional quality of longitudinal follow-up. We identified individuals who exhibit MTB-specific T cell responses, have not undergone preventive treatment, and yet have not developed active tuberculosis over the subsequent years to decades.^6^

The SHCS also serves as an ideal cohort for investigating natural immunity to TB and the effects of antiretroviral treatment, offering valuable insights into the MTB aspect of HIV-1/MTB coinfection. While our analysis primarily focuses on viral control in PWH due to the distinct phenotype observed in MTB-infected patients^9^ and reveals surprising and underexplored outcomes of MTB infection with therapeutic potential, we also conducted a preliminary analysis of MTB signatures independent of HIV-1 viremia. Our initial results suggest a higher baseline reactivity to interferon and the absence of the anergic phenotype typically linked to HIV-1-related TB (Figure S6, related to Figures 1 and 2), which may account for the lower incidence of active TB in these individuals over the long term, but further investigation is needed to confirm these observations.

While numerous investigations have delved into the examination of natural immunity to HIV-1 through comparisons of elite controllers and long-term non-progressors,^24^ establishing definitive causality has remained a formidable challenge. While our current findings do not conclusively establish causation either, our methodology represents a step forward by delineating transcriptional perturbations associated with MTB infection in the absence of HIV-1 viremia. We then systematically explored the persistence of these perturbations during HIV-1 viremia, both at the level of pathway enrichment, individual gene expression level and if used for a signature score.

In aggregate we propose that, in specific individuals exposed to MTB, the heightened inflammation does not unequivocally translate into active disease. Instead, our findings suggest that in a subset of patients, the activated innate immune system is correlated with enhanced control of HIV-1. Consequently, our data seamlessly align with the evolving idea of MTB infection as a spectrum of diseases, wherein stages of MTB infection differently influence the innate immune system.

### Limitations of the study

Our cohort includes only PWH selected from a longitudinal cohort study in Switzerland, which may introduce various selection biases. Additionally, while our bulk RNA sequencing approach provides a comprehensive overview, it may overlook subtleties that single-cell sequencing could reveal. Also, our findings warrant careful consideration due to the limited number of individual patients involved. However, it is noteworthy that we have attempted to address this limitation through meticulous patient matching, a factor that enhances the robustness of our study. Despite the small cohort, a consistent signal emerges associated with MTB-infected patients. This observed consistency across a limited number of cases underscores the potential significance of the signal, suggesting that even in the context of limited patient numbers, the impact of MTB infection is noteworthy and warrants further investigation.

In our study, and in line with our previous investigation,^9^ we defined MTB infection based on clinical standards, using either a 5 mm TST or a positive IGRA result. This approach might introduce a bias, as the TST is generally less specific than the IGRA. However, it is debatable how significant these differences are in PWH, as most studies have not found a convincing benefit of IGRA over TST in this immune-compromised population.^25^^,^^26^^,^^27^^,^^28^

## Resource availability

### Lead contact

Further information and requests for resources and reagents should be directed to and will be fulfilled by the lead contact, Johannes Nemeth (johannes.nemeth@usz.ch).

### Materials availability

This study did not generate new unique reagents.

### Data and code availability

• Data: The SHCS informed consent states that sharing data outside the SHCS network is only permitted for specific studies on HIV infection and its complications, and to researchers who have signed an agreement detailing the use of the data and biological samples.
• Code: Code is based on the SHCS data, therefore not open to sharing outside the SHCS network.
• All other items: Any additional information required to reanalyze the data reported in this paper is available from the lead contact upon request.

## Acknowledgments

We would like to thank all the SHCS study participants, doctors and nurses.

Funding information: This study was supported by the 10.13039/501100001711Swiss National Science Foundation (SNF) grant 310030_200407, the Theodor und Ida Herzog Stiftung, SHCS Project Funding 857 and the 10.13039/100000002NIH Grant U19AI100627. The SHCS is funded by different sources, mainly by the 10.13039/501100001711Swiss National Science Foundation (SNSF), companies and the 10.13039/501100010474Swiss HIV Cohort Research Foundation. Since 2000, the Swiss National Science Foundation (SNSF) has been the major funding organization of the SHCS.

## Author contributions

Conceptualization, B.T., L.J., C.D.S., and J.Nemeth; methodology, B.T., L.J., C.D.S, and J.Nemeth.; investigation, B.T., L.J., C.D.S., L.O., J.T., R.W., K.N., F.D., and J.Nemeth.; writing—original draft, B.T., L.J., J.Nemeth.; writing—review and editing, B.T., L.J., G.O., K.J.M., D.L.B., H.F.G., R.D.K., F.D., and J.Nemeth.; funding acquisition, L.J. and J.Nemeth.; resources, L.O., K.K., M.Z., L.B., M.K., J.Notter., H.F., M.H., H.H.H., A.C., M.C., N.D.L., E.B., D.L.B., and H.F.G.; supervision, K.J.M., H.F.G., R.D.K., and J.Nemeth.

## Declaration of interests

A.C. received grants from Merck Sharp & Dohme (MSD), ViiV Healthcare, and Gilead Sciences for unrelated research. R.D.K. received grants from Gilead Sciences and National Institutes of Health (NIH) for unrelated research. D.L.B. received honoraria for working on the advisory board of Gilead Sciences, Merck, ViiV, Pfizer, and AstraZeneca. D.L.B. received honoraria for presentations from Gilead Sciences and Merck. E.B. received grants from MSD for unrelated research. E.B. received payments for travel reimbursement from ViiV, MSD, Gilead Sciences, Pfizer, and Abbvie. E.B. received honoraria for working on the advisory board of ViiV, MSD, Pfizer, Gilead Sciences, AstraZeneca, and Ely Lilly. H.H.H. received honoraria for working on the advisory board of AiCuris, Merck, Vera Dx, and Molecular Partners. H.H.H. received honoraria for presentations from Merck, Gilead Sciences, Biotest, and Vera Dx. J.N. received honoraria for presentations from Oxford Immunotec, Gilead and ViiV. H.FG. received honoraria for working on the advisory board of Gilead Sciences, Merck, ViiV, Janssen, Johnson and Johnson, Novartis, and GlaxoSmithKline (GSK). H.F.G. received payments for travel reimbursements from Gilead Sciences. H.F.G. received grants from NIH, Yvonne Jacob Foundation, and Gilead Sciences.

## STAR★Methods

### Key resources table

**Table:** 

REAGENT or RESOURCE	SOURCE	IDENTIFIER
**Antibodies**		

NIR Zombie dye APC-Cy7	Biolegend	Cat No. 423105
CD20 PE	Biolegend	Cat No. 302306
CD3 AF700	Biolegend	Cat No. 300324
CD69 FITC	Biolegend	Cat No. 310904
CD56 BV510	Biolegend	Cat No. 304608
CD16 PE-Dazzle	Biolegend	Cat No. 302054
HLA-DR BV605	Biolegend	Cat No. 307640
CD11b PE-Cy7	Biolegend	Cat No. 301322
CD14 BV421	Biolegend	Cat No. 301829

**Chemicals, peptides, and recombinant proteins**		

PMA/Ionomycin	ThermoFischer	00-4975-93
Cytofix/Cytoperm buffer	BD Biosciences	Cat No. 554722

**Critical commercial assays**		

RNeasy Micro Kit (50)	Qiagen	Cat. No./ID: 74004
1 × 100 bp single-end read sequencing kit SmartSeq2 library preparation (20ng total RNA input, minimum read depth 30 million reads per sample)	Illumina	

**Experimental models: Cell lines**		

No cell lines were used		

**Software and algorithms**		

R Software		
FlowJo version 10.0.8r1		
TapeStation	Agilent	
NovaSeq6000	Illumina	

### Experimental model and study participant details

#### Selection and matching of study population

We performed a nested, retrospective case-control matched study investigating patients at the time point of MTB infection diagnosis and sourced peripheral blood mononuclear cells (PBMC) from 36 people living with HIV (PWH) participating in the Swiss HIV Cohort Study (SHCS).^13^ For each patient, one sample (0.2 × 10^6^ cells) was retrieved at the time of MTB screening. The SHCS is a prospective, longitudinal, multicenter, representative cohort study that collects demographic and clinical data from people with HIV-1 twice a year and stores plasma and PBMC samples in a biobank.

We wrote a matching function in R calculating the distance of each patient to the rest of the cohort given their sex, ethnicity, and CD4 counts; and then optimizing the choice of pairing by minimizing this distance for each patient (Figure S2, related to Tables 1 and 2).

MTB testing (either TST or IGRA) was only performed once at the inclusion date. None of the individuals developed active TB during the follow up. Details on ethical board review, informed consent and overall cohort structure are published in the SHCS cohort profile.^13^

MTB positivity was defined based on one or more positive MTB infection tests (IGRA, TST, or both) in the absence of diagnosed pulmonary or extrapulmonary TB. We selected only those with unequivocal TB test results. These samples were then categorized into four groups based on their *Mycobacterium tuberculosis* infection (MTB) and HIV infection status: *i)* MTB positive and viremic (MTB+ HIV^vir^; *n* = 7), *ii)* MTB positive and suppressed (MTB+ HIV^supp^; *n* = 11), *iii)* MTB negative and viremic (MTB- HIV^vir^; *n* = 7), *iv)* MTB negative and suppressed (MTB- HIV^supp^; *n* = 11). For each sample, we used the TB test closest to the time of blood collection. PWH were classified as suppressed if their HIV viral load was below 500 copies/ml and as viremic if it was above or equal to this threshold. As outlined in Table 1, all patients detected by the algorithm had an undetectable HIV-1 viral load. We included only samples collected post-2000 to minimize the risk of cell degradation. Our selection was also influenced by practical constraints, such as the availability of sample aliquots and the viability of PBMCs. Individuals with opportunistic infections were excluded (detailed list in Table S2, related to Tables 1 and 2).

We considered sex, ethnicity, and CD4 cell counts when matching MTB- and MTB+ individuals within each HIV status group. Matching process involves first identifying all potential matches with the same sex and ethnicity, then selecting the individual with the closest CD4 cell count. Once a pair was matched, they were removed from the pool to prevent duplication in the matching process (Figure S2, related to Tables 1 and 2).

### Method details

#### RNA isolation and sequencing

After thawing, 0.2 × 10^6^ cells were cultivated for 6 h in RPMI. For the positive control, cells were incubated with 150 ng/mL PMA/Ionomycin. Following cell stimulation, the samples were centrifuged for 5 min at 600 g. RNA isolation was then performed using the RNeasy Micro Kit (Qiagen), following the manufacturer’s instructions. This process was carried out either manually or utilizing the QIAcube (Qiagen). The quantity of isolated RNA was assessed on a TapeStation (Agilent) employing a high-sensitivity RNA ScreenTape. For RNA sequencing (RNAseq), 20 ng of total RNA was used as input. We utilized the SmartSeq2 library preparation method, and sequencing was conducted with a 1 × 100 bp single-end read configuration (Illumina). Each sample was sequenced to a minimum depth of 30 million reads using the NovaSeq6000 system (Illumina), according to the manufacturer’s guidelines. All sequencing was performed at the Functional Genomics Centre Zurich (FGCZ).

#### Flow cytometric analysis

For flow cytometric analysis, cells underwent two washes with PBS to eliminate any remaining stimulation compounds. Surface proteins were then stained at 4°C for 30 min using the following markers: NIR Zombie dye APC-Cy7 (Biolegend, Cat No. 423105), CD20 PE (Biolegend, Cat No. 302306), CD3 AF700 (Biolegend, Cat No. 300324), CD69 FITC (Biolegend, Cat No. 310904), CD56 BV510 (Biolegend, Cat No. 304608), CD16 PE-Dazzle (Biolegend, Cat No. 302054), HLA-DR BV605 (Biolegend, Cat No. 307640), CD11b PE-Cy7 (Biolegend, Cat No. 301322), and CD14 BV421 (Biolegend, Cat No. 301829). Post-staining, the cells were washed again with PBS and fixed using Cytofix/Cytoperm buffer (BD Biosciences, Cat No. 554722) for 20 min at 4°C. Following another wash, cells were resuspended in PBS. The flow cytometry was conducted on an LSR II Fortessa 4L equipped with a High throughput sampler (HTS) (Figure S4, related to Figures 1 and 2). Data analysis was performed using FlowJo version 10.0.8r1 and R software.

### Quantification and statistical analysis

#### RNAseq analysis

The RNA sequencing raw reads were first cleaned by removing adapter sequences, trimming low quality ends, and filtering reads with low quality (phred quality <20) using Fastp (Version 0.20).^15^ Sequence pseudo alignment of the resulting high-quality reads to the human reference genome (build GRCh38.p13) and quantification of gene level expression (gene models based on GENCODE release 37) was carried out using Kallisto (Version 0.46.1).^16^ Differential expression was computed using the raw counts obtained from Kallisto and a generalized linear model as implemented in the Bioconductor package DESeq2 (R version: 4.2.0, DESeq2 version: 1.36.0).^17^ Genes showing altered expression with adjusted (Benjamini and Hochberg method) *p*-value <0.05 were considered differentially expressed. Batch effects were corrected using the removeBatchEffect() function from the limma package (v3.54.1).^18^ Due to technical issues, certain samples were not successfully sequenced. These samples included 2 matched MTB−/+ pairs (4 samples) belonging to HIV^supp^, 2 samples belonging to MTB- HIV^vir^, and 1 sample belonging to MTB+ HIV^vir^ from the untreated pool (negative control). Consequently, this lowered the number of matched samples from 11 to 9 for HIV^supp^, and led to an unmatched HIV^vir^ population with 5 MTB- and 6 MTB+ samples. Given the already low number of samples available, for the dimensionality reduction and clustering analysis, we proceeded with the unmatched HIV^vir^ population since the MTB−/+ is a binary categorization. However, this mismatch was a bigger concern when calculating the viral load correlations. Therefore, for every sample that failed sequencing, its corresponding matched sample was also excluded, resulting in a final count of 4 matched pairs for HIV^vir^. Dimensionality reduction and clustering analysis is performed on this matched population as well, given in Figure S5, related to Figure 2.

#### Dimensionality reduction and clustering analysis

K-means clustering was applied to normalized gene counts using the k-means function in the R stats package. We focused on genes with a differential expression *p*-value below 0.01. The number of clusters was set to four, corresponding to the four sample groups for each HIV phenotype (unstimulated MTB+, stimulated MTB-, stimulated MTB+ with PMA/Ionomycin, and stimulated MTB- with PMA/Ionomycin).

For our Gene Set Enrichment Analysis (GSEA), we utilized the Hallmark database.^19^ When visualizing the heatmaps, *Z* score normalization was applied to the log2-transformed normalized read counts for each gene.

#### Correlation calculations between viral load and gene expression values

We analyzed the correlation between log10-transformed viral RNA counts and log10-transformed FPKM values, focusing on conserved and non-conserved transcripts with criteria of abs (LogFC) > 1 and *p*-value<0.01. The distributions of these correlation coefficients were visualized in boxplots, and a one sided t-test was used to determine whether the non-conserved transcripts had a significantly greater correlation value.

Permutation tests were conducted by creating 1,000 random subsets, each matching the size of our conserved transcript gene pool, drawn from the entire transcript set. For each subset, average FPKM values were computed, taking into account the direction of gene regulation. These values were then correlated with log10-transformed viral RNA counts to assess the significance of our observed correlations for the conserved transcripts.

#### Integration and comparison to other datasets

To validate our results, we utilized an independent dataset: a cohort comprising PWH with either MTB infection or active TB (PWH MTB+, PWH developing active TB).^17^

The dataset from Kaforou et al.^17^ provided background-subtracted, quantile-normalized whole blood microarray expression data (downloaded from NCBI GEO, GSE37250). We applied a log2 transformation to this data. Signature scores^21^ were calculated per patient by subtracting the expression level of the downregulated genes from the upregulated genes in MTB+ compared to MTB-, given the direction of regulation determined in our study. We applied one-tailed t-tests to compare the distribution of signature scores statistically.
