# Supplementary material for: Transcriptional profile of Mycobacterium tuberculosis infection in people living with HIV
Source: iScience. 2024 Oct 21;27(11):111228. doi: 10.1016/j.isci.2024.111228 (PMC11565417; doi:10.1016/j.isci.2024.111228)
Supplement: Document S1. Figures S1–S6 and Tables S1 and S2 [file mmc1.pdf]

iScience, Volume *27*

Supplemental information

Transcriptional profile of *Mycobacterium tuberculosis* infection in people living with HIV

Burcu Tepekule, Lisa Jörimann, Corinne D. Schenkel, Lennart Opitz, Jasmin Tschumi, Rebekka Wolfensberger, Kathrin Neumann, Katharina Kusejko, Marius Zeeb, Lucas Boeck, Marisa Kälin, Julia Notter, Hansjakob Furrer, Matthias Hoffmann, Hans H. Hirsch, Alexandra Calmy, Matthias Cavassini, Niklaus D. Labhardt, Enos Bernasconi, Gabriela Oesch, Karin J. Metzner, Dominique L. Braun, Huldrych F. Günthard, Roger D. Kouyos, Fergal Duffy, Johannes Nemeth, and the Swiss HIV Cohort Study

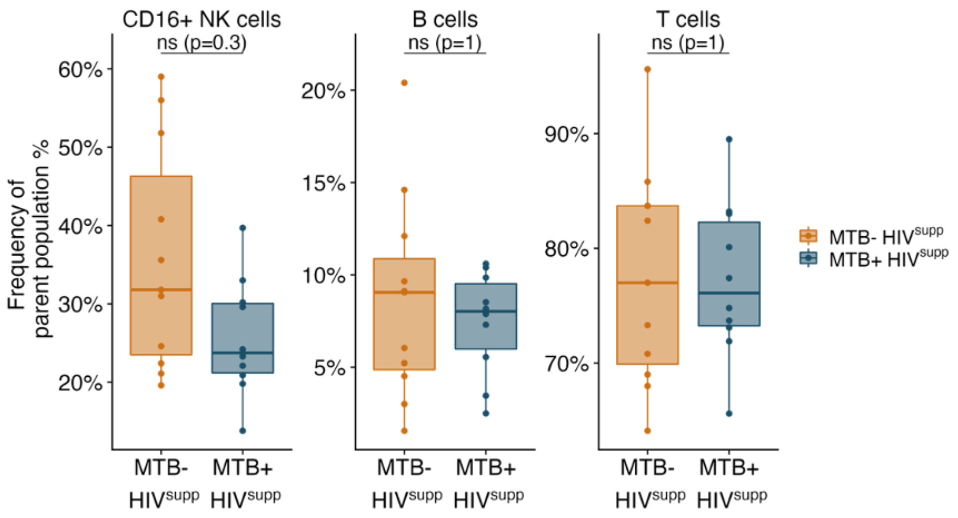

Fig. S1a. Flow cytometry analysis for HIV^{supp} individuals, related to Figure 1. Frequency of CD16+ NK cells, B cells and T cells in MTB+ HIV^{supp} (blue) and MTB- HIV^{supp} (orange) after flow cytometry analysis. Shown are boxplots with median and standard deviation. Wilcoxon test is shown for each cell type (CD16+ NK cells: p=0.3, B cells: p=1, T cells: p=1). CD16+ NK cell (MTB+ HIVsupp: 1.62 (1.14, 1.97), MTB- HIVsupp: 2.13 (1.05, 4.16), median (IQR), p=0.3), B cell (MTB+ HIVsupp: 4.55 (3.32, 5.46), MTB- HIVsupp: 4.42 (3.22, 6.97), median (IQR), p=1) or T cell (MTB+ HIVsupp: 50.5 (36.6, 59.8), MTB- HIVsupp: 55.7 (48.4, 58.4), median (IQR), p=1)

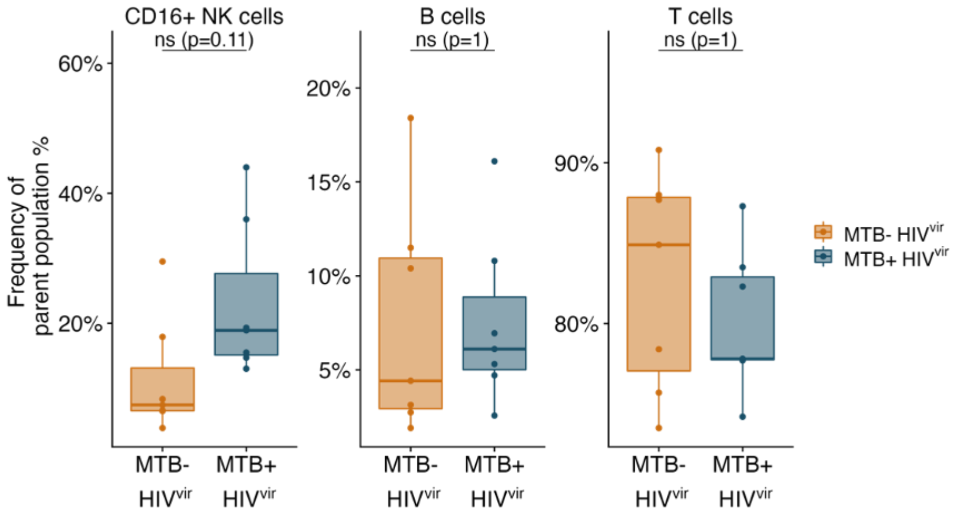

Fig. S1b. Flow cytometry analysis for HIV^{vir} individuals, related to Figure 2. Frequency of CD16+ NK cells, B cells and T cells in MTB+ HIV^{vir} (blue) and MTB- HIV^{vir} (orange) after flow cytometry analysis. Shown are boxplots with median and standard deviation. Wilcoxon test is shown for each cell type (CD16+ NK cells: p=0.11, B cells: p=1, T cells: p=1).

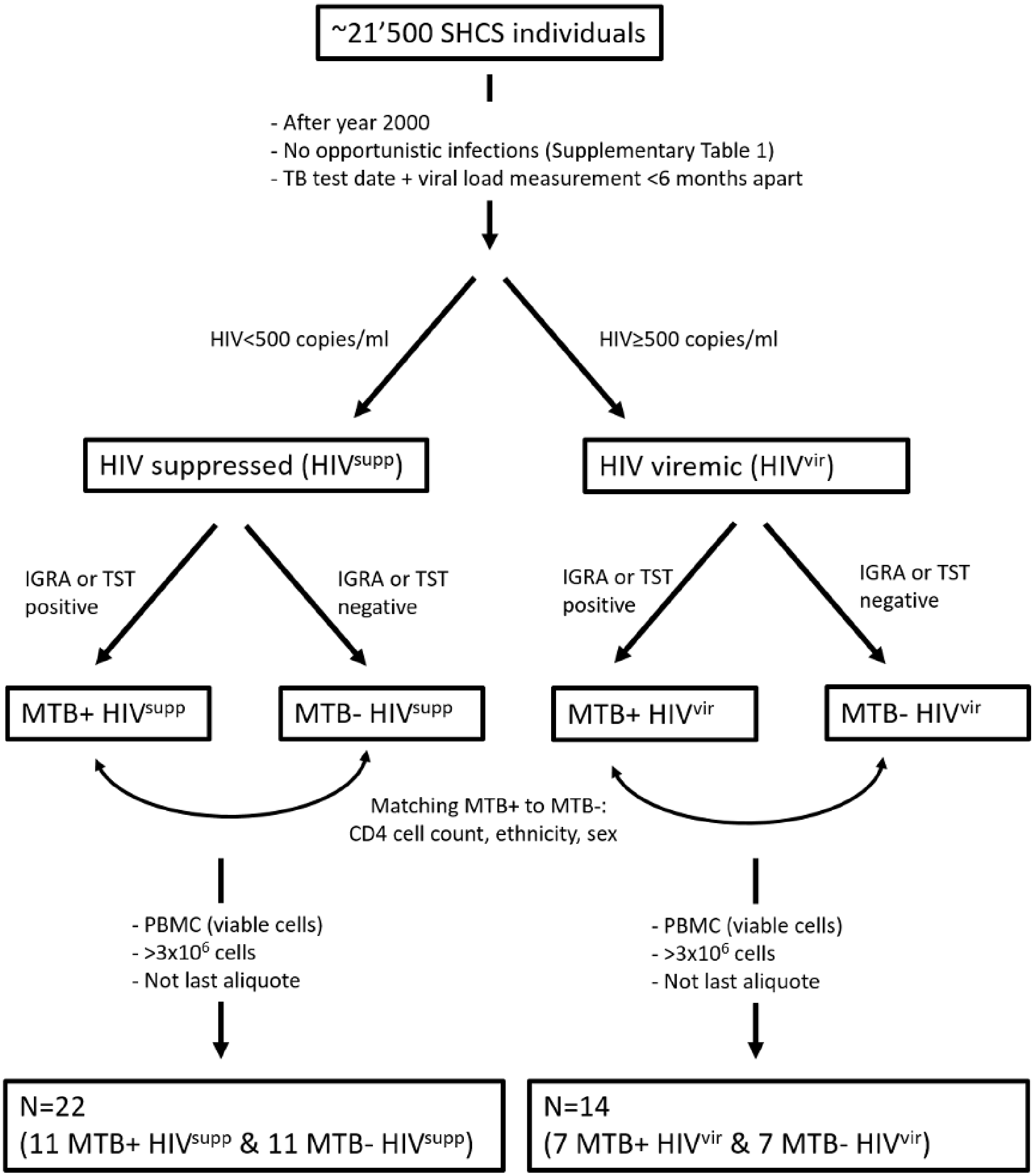

Fig. S2. Flow chart of selection criteria for PWH, related to STAR Methods. Shown are number of individuals after each selection step and the corresponding selection criteria.

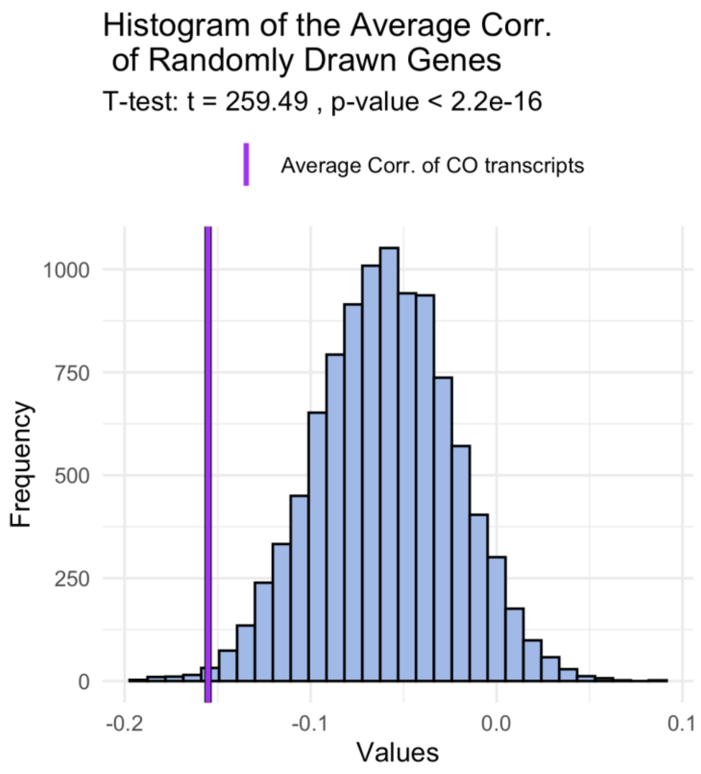

Figure S3 Permutation test results, related to STAR Methods. Histogram of the average correlation between HIV-1 viral load and the expression levels for each randomized set generated during the permutation test.

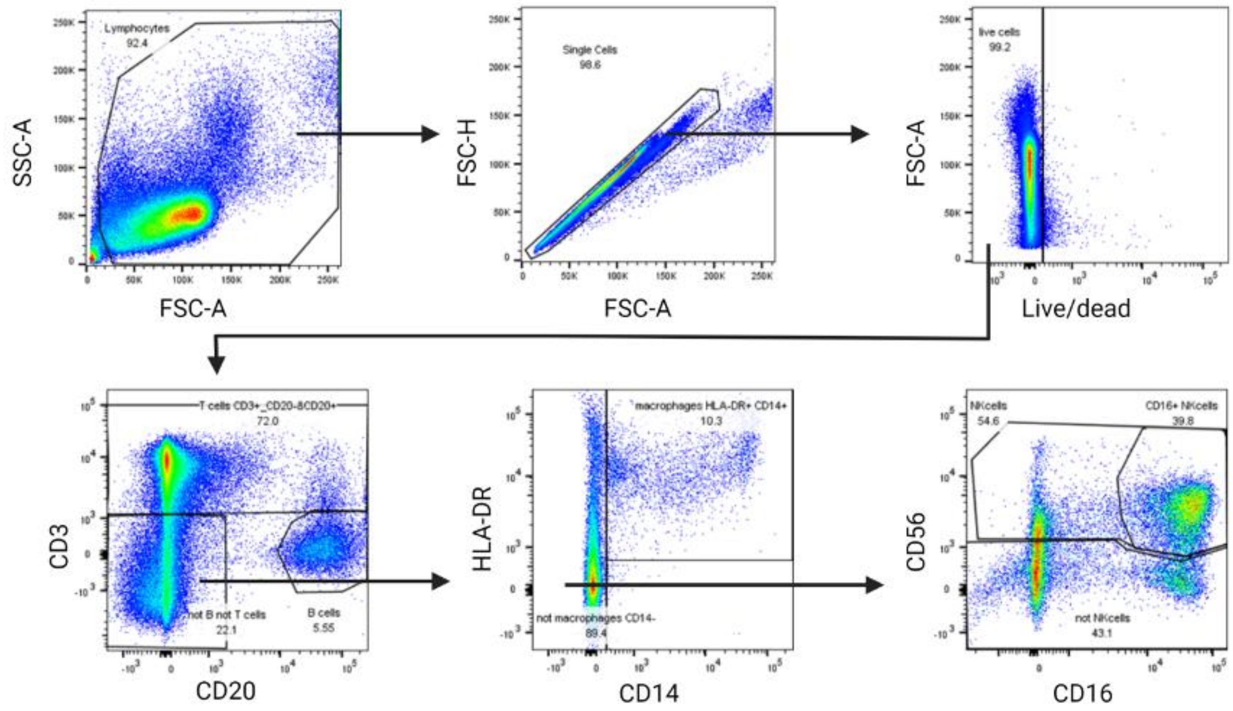

Fig. S4. Gating strategy for flow cytometry analysis, related to STAR Methods. PBMCs from PWH were obtained and live cells were gated on CD3 and CD20. CD3-/CD20- cells were further gated on HLA-DR and CD14 and CD14- cells were further gated on CD56 and CD16.

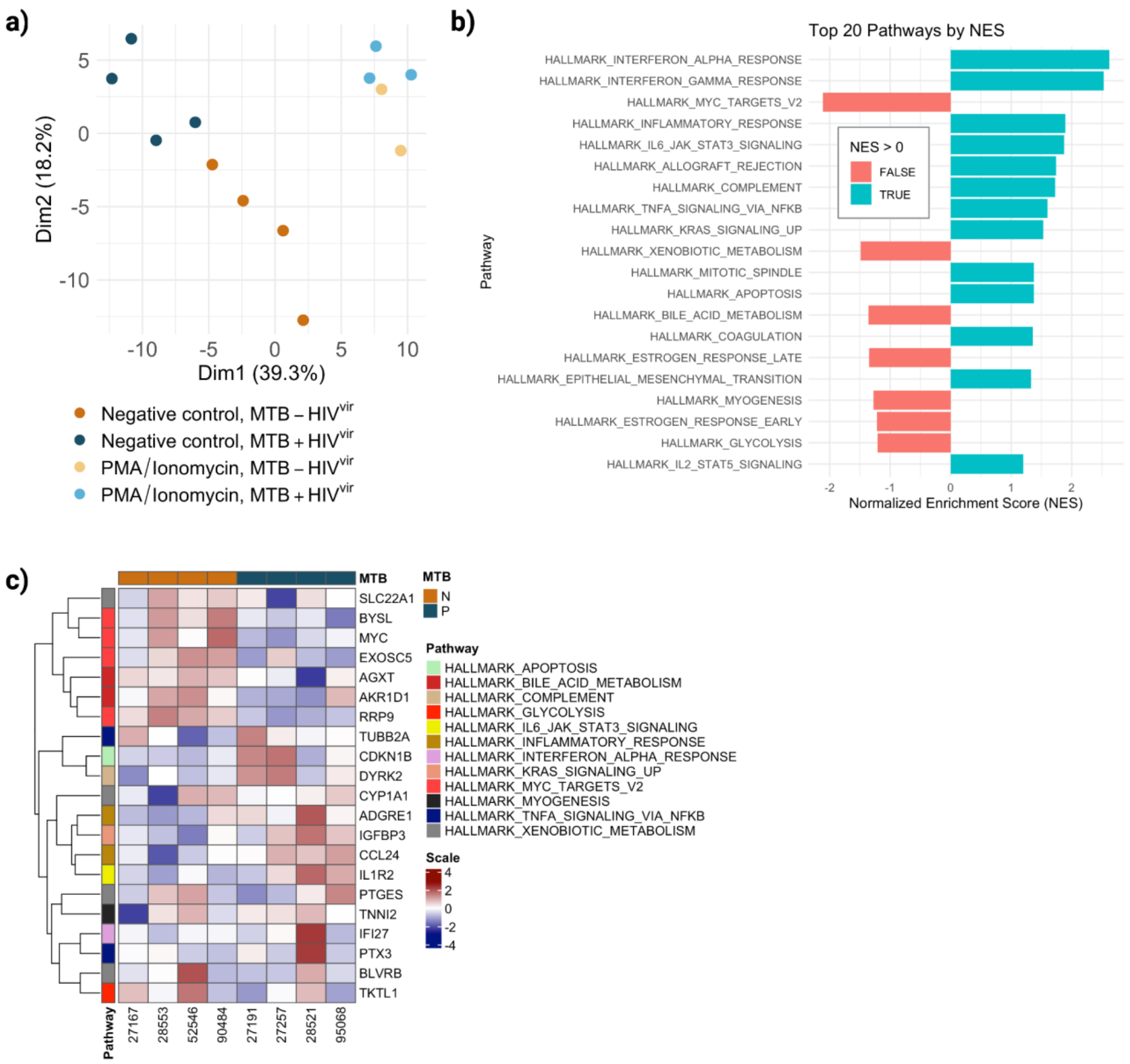

Fig. S5. MTB Perturbations in viremic PWH, including only 4 matched pairs for HIV^{vir}, related to Figure 2. a) Dimensionality reduction and clustering analysis for HIV-viremic individuals with and without MTB infection (MTB+ HIV^{vir} and MTB- HIV^{vir}). **b)** First 20 pathways with the highest normalized enrichment score (NES) based on the gene-set enrichment analysis (GSEA). **c)** Heatmap displaying the leading edge genes of these pathways filtered by p-value <0.01. The color scale ranges from dark blue (representing downregulated genes) to dark red (representing upregulated genes), with lighter tones indicating intermediate values. Rows represent genes, and columns represent samples, annotated by MTB status (N for negative, P for positive). The data are log2-transformed and clustered by genes. The scale bar indicates log2 expression levels ranging from -4 to 4.

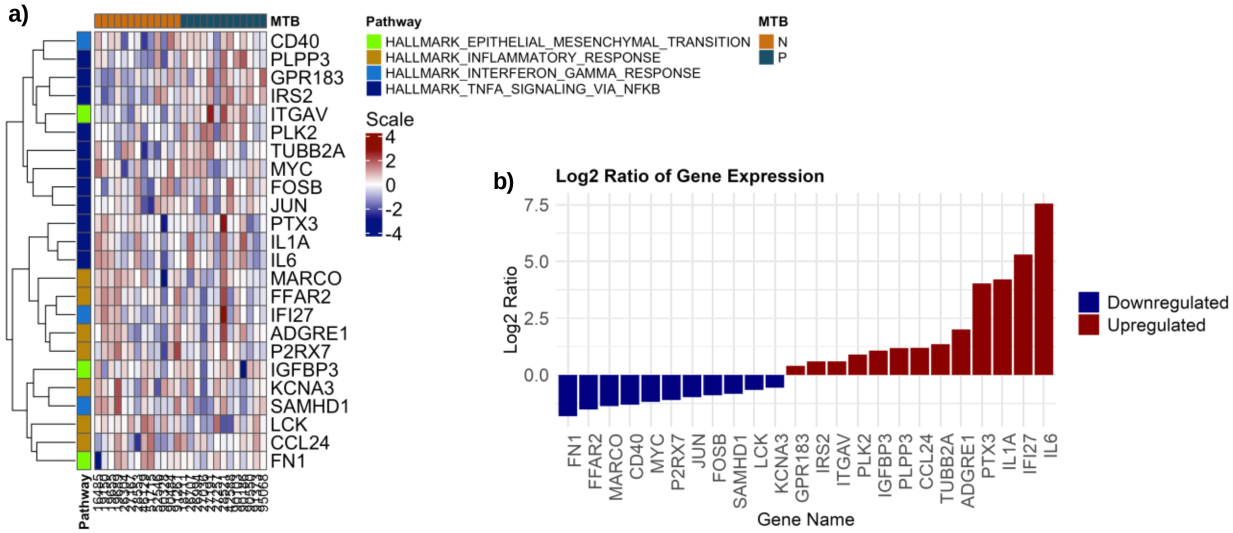

Fig. S6. MTB Perturbations regardless of viremia, related to Figure 1, Figure 2. a) Heatmap displaying the leading edge genes of the first 4 pathways with the highest normalized enrichment score (NES) based on the gene-set enrichment analysis (GSEA) pathways filtered by p-value <0.01. **b)** Fold change (Log2 ratios) of the differentially expressed genes. Data is pooled for HIV^{vir} and HIV^{supp} individuals, highlighting the transcriptional changes induced by the MTB infection regardless of the HIV infection status.

Table S1. List of carried over transcripts, related to Figure 3. List of carried over transcripts with their corresponding names, log fold change values, p values, and correlation with the viral load.

gene_name	log2_HIVP	pValue_HIVP	log2_HIVN	pValue_HIVN	corr_FPKM
AMY2B	1.498517	0.005402	0.053979	0.774175	-0.706108
TUBB2A	1.356838	0.003883	0.255625	0.529333	-0.648773
AC008581.2	3.607487	0.001657	0.935091	0.135618	-0.597054
CEACAM21	1.55689	0.000532	0.021003	0.947182	-0.566616
TUBB2B	4.28338	0.000159	0.200812	0.796855	-0.477471
NEO1	2.097385	0.000366	0.252594	0.486511	-0.474355
GNLY	2.202992	0.001251	0.084538	0.875132	-0.422873
SFTPB	-1.090287	0.005174	-0.14842	0.659321	-0.417074
WWC2	1.552436	0.003687	0.314738	0.119743	-0.409493
BX664615.2	2.844257	0.000224	0.378037	0.392991	-0.364135
FAM156A	-1.516044	0.007314	-0.468915	0.175433	-0.363551
AC010616.1	2.198713	0.001406	0.220761	0.738423	-0.308294
RAB34	-1.5222	0.001228	-0.479224	0.248506	-0.287333
PDE4C	-1.035886	0.001394	-0.155513	0.597327	-0.275278
IGFBP3	1.07467	0.003701	0.166862	0.78135	-0.228854
AP000295.1	7.895425	0.005833	1.313258	0.182597	-0.152229
XAB2	1.204309	0.003235	0.805784	0.059978	-0.125595
SNED1	1.550061	0.009726	0.570657	0.080132	-0.087102
HS3ST3B1	2.105495	0.007716	0.630545	0.190118	-0.008914
STAC2	-1.013961	0.006512	-0.126949	0.697539	-0.004616
AKR1D1	-1.175365	0.007009	-0.370139	0.276529	0.053803
IGKV3-7	-2.051104	0.002957	-0.816776	0.433453	0.054796
CDCA8	1.419941	0.003837	0.167595	0.567989	0.070205
AC020915.4	5.249703	0.000122	0.308257	0.611476	0.075333
AC018695.7	-1.325224	0.00835	-0.294461	0.386277	0.106546
CYP1A1	-2.043803	0.00491	-0.302491	0.660909	0.137494
IGHV4-59	-1.563449	0.003276	-0.468012	0.310715	0.264127
RERG	-1.500063	0.005628	-0.703857	0.065624	0.267226
IGHV1-69D	1.561447	0.004855	0.434493	0.598272	0.279949
IGKV1-12	-1.733855	0.001667	-0.388062	0.581132	0.285719
CCBE1	-1.300812	0.003055	-0.026809	0.934125	0.298944
SIGLEC6	-1.430166	0.00124	-0.036194	0.901716	0.332201

Table S2. List of opportunistic infections that were excluded for selection of PWH, related to STAR Methods

Aids defining disease not specified
Bacterial pneumonia, recurrent
Cytomegalovirus (CMV) - retinitis
Cytomegalovirus (CMV) disease, other
Candidiasis of trachea, bronchi or lungs
Candidiasis, oesophagial
Carcinoma, cervical, invasive
Coccidioidomycosis disseminated
Cryptococcal meningitis
Cryptococcosis, other disseminated
Cryptosporidiosis, Diarrhea > 1 month
Encephalopathy, HIV-related
Herpes simplex disease, visceral
Histoplasmosis disseminated
Intracerebral lesions, indeterminate
Isosporiasis , Diarrhoe > 1 month
Kaposi sarcoma
Mycobacterium avium - intracellulare, disseminated
Mycobacterium genavense disease
Mycobacterium kansasii disease
Mycobacterium avium complex or kansasii
Mycobacterium other species disseminated or extrapulmonary
Non-Hodgkin's lymphoma
Pediatric category C disease
Pneumocystis disease, extrapulmonary
Pneumocystis pneumonia
Salmonella septicemia, recurrent
Toxoplasmosis disseminated
Toxoplasmosis, cerebral
Tuberculosis pulmonary
Wasting Syndrome, AIDS-defining
